# Observing the Temperature Dependent Transition of the GP2 Peptide Using Terahertz Spectroscopy

**DOI:** 10.1371/journal.pone.0050306

**Published:** 2012-11-28

**Authors:** Yiwen Sun, Zexuan Zhu, Siping Chen, Jega Balakrishnan, Derek Abbott, Anil T. Ahuja, Emma Pickwell-MacPherson

**Affiliations:** 1 National-Regional Key Technology Engineering Laboratory for Medical Ultrasound, Guangdong Key Laboratory for Biomedical Measurements and Ultrasound Imaging, Department of Biomedical Engineering, School of Medicine, Shenzhen University, Shenzhen, China; 2 College of Computer Science and Software Engineering, Shenzhen University, Shenzhen, China; 3 Centre for Biomedical Engineering and School of Electrical and Electronic Engineering, University of Adelaide, Adelaide, Australia; 4 Diagnostic Radiology and Organ Imaging Department, The Chinese University of Hong Kong, Shatin, Hong Kong; 5 Department of Electronic Engineering, Chinese University of Hong Kong, Shatin, Hong Kong; Russian Academy of Sciences, Institute for Biological Instrumentation, Russian Federation

## Abstract

The GP2 peptide is derived from the Human Epidermal growth factor Receptor 2 (HER2/nue), a marker protein for breast cancer present in saliva. In this paper we study the temperature dependent behavior of hydrated GP2 at terahertz frequencies and find that the peptide undergoes a dynamic transition between 200 and 220 K. By fitting suitable molecular models to the frequency response we determine the molecular processes involved above and below the transition temperature (*T*_D_). In particular, we show that below *T*_D_ the dynamic transition is dominated by a simple harmonic vibration with a slow and temperature dependent relaxation time constant and that above *T*_D_, the dynamic behavior is governed by two oscillators, one of which has a fast and temperature independent relaxation time constant and the other of which is a heavily damped oscillator with a slow and temperature dependent time constant. Furthermore a red shifting of the characteristic frequency of the damped oscillator was observed, confirming the presence of a non-harmonic vibration potential. Our measurements and modeling of GP2 highlight the unique capabilities of THz spectroscopy for protein characterization.

## Introduction

Proteins play a critical role in biological processes and often require an aqueous phase to be transported to their target sites. A variety of experiments have demonstrated that proteins influence both the spatial and dynamic arrangement of neighboring liquid layers through weak intermolecular interactions [1]. This dynamical process can be considered as being described by collective vibrational modes, individual bond vibrations and determined by the energy necessary for motion relative to the ambient temperature [2]. This temperature (∼200 K for water based solvents) is known as the dynamical transition temperature (*T*_D_). Several papers have focused on the prediction of the dynamic transition in hydrated macromolecules using different techniques, including neutron scattering, Mössbauer spectroscopy, and X-ray diffraction [3]–[5]. It is widely believed that this temperature is affected by many factors, e.g. molecular weight [6], [7], bond interactions [8], polar groups [9] and backbone flexibility [10], furthermore different results have been reported for the same protein type depending on the detection method [8], [10]. However, this dynamical phenomenon has not been intensely studied at terahertz (THz) frequencies and motivates further fundamental research.

Research shows that the HER2/neu protein is a prognostic breast cancer marker [11] assayed in tissue biopsies from women diagnosed with malignant tumors. HER2/neu (also known as c-erbB-2) stands for “Human Epidermal growth factor Receptor 2”. The HER2/neu protein is over-expressed in about 20–30% of malignant breast tumors and has been used in postoperative follow-up evaluation as an indicator of patient relapse [11]. GP2 (HER2/neu, 654–662, IISAVVGIL) is a nine amino acid peptide derived from HER2/neu (654–662) with amino acid sequence: {ILE}{ILE}{SER}{ALA}{VAL}{VAL}{GLY}{ILE}{LEU}. It has been identified using tumor-associated lymphocytes isolated from patients with ovarian and breast cancers [12] and a GP2 peptide vaccine is currently being evaluated in a phase II efficacy trial enrolling breast cancer patients [13].

Various authors have predicted that proteins have vibrational resonances in the THz frequency range [14], [15] and THz time domain spectroscopy pulses are ideally suited to probing picosecond and subpicosecond transient behaviors of hydrated proteins. The dielectric spectrum is sensitive to the molecular polarization so THz spectroscopy can often reveal subtle changes in larger molecules such as cis-trans forms, which can be used for biomedical diagnostics [16]–[18]. Indeed, we have demonstrated that the THz dielectric spectrum is sensitive to the conformation of proteins in hydrogen-bonded networks [19], [20].

Based on thermal radio theory, all particles are in their positional minimum at thermal equilibrium [21]. When a protein solution is cooled down and the molecules do not reach their energetically preferred point, at a certain temperature, the substance enters dynamic arrest and becomes disordered. This anomalous phenomenon is called a dynamical transition [22]. In neutron scattering measurements, hydrated proteins undergo their dynamical transitions at a particular temperature, which is characterized by a sharp deviation from linearity of the temperature dependence of the amplitude of anharmonic dynamics [23]. Many papers have reported that the dynamic temperature (*T*_D_) is dependent on the type of protein (e.g. molecular weight, bond interactions, polar groups and backbone flexibility). Furthermore, *T*_D_ is also dependent on the frequency range of the measurement technique. For instance the *T*_D_ of hydrated lysozyme was estimated to be 180 K using light (Raman and Brillouin) scattering spectroscopy [24], whereas Markelz et al. found that THz measurements of hen egg white lysozyme underwent a dynamical transition at about 200 K [25].

Significantly, HER2/neu is present in saliva so detecting and determining the properties of this protein in its hydrated form may be of future diagnostic value for breast cancer detection. In this paper we present a fundamental study of hydrated GP2 using THz time domain spectroscopy. Our aim is to find how *T*_D_ of GP2 is dependent on frequency and determine the underlying mechanisms behind the frequency dependence. We will use a classical Cole-Davison model and our newly devised Cole-Davison-Resonant-Absorption (CDRA) model to describe the interactions between the peptide and its solvent molecules below and above *T*_D_ respectively. Further studies with appropriate controls could potentially lead to a convenient THz cancer test via the saliva and this underlies the motivation for the present fundamental study.

## Methods

The molecular formula of GP2 is C_42_H_77_N_9_O_11_ and the molecular weight is 884.12 Da. The GP2 powder (GenScript Corporation, Piscataway, USA) was dissolved into pH 7.0 buffer at a concentration of 20 mg/ml. The buffer, composed of 3.54 g potassium dihydrogen phosphate and 14.7 g disodium hydrogen phosphate per liter, was purchased from Sigma-Aldrich (USA). Although the concentration of 20 mg/ml is much higher than that present in human saliva, this is a fundamental study to identify any potential terahertz features for further investigation. The sample holder was fabricated from TOPAS™ 5013L-10, this material (which is a Cyclic Olefin Copolymer) was chosen as it has very low attenuation at THz frequencies [26], [27]. The hydrated protein formulation was pipetted into the sample holder for the THz measurement. The clean homogeneous empty sample cell was also measured as a reference (refractive index ∼1.53) enabling the spectroscopic properties of the sample to be accurately determined. An optical cryostat (Oxford Instruments 9146) was also used to cool the sample for measurement in the temperature range *T* = 15–294 K. The main measurements were carried out by decreasing the temperature from *T* = 294 K, the sample was equilibrated for at least 30 minutes at each temperature. A typical transmission mode THz time domain spectroscopy system with useable bandwidth 0.1–1.5 THz was used to obtain the THz spectra. Three samples were measured 10 times at 8 different temperatures during the cooling process. Additionally a few temperature points were measured on heating the samples back to room temperature and no significant changes in the measured spectra were observed. This indicated reproducibility of the data and no loss in hydration level of the sample during the measurements.

## Results and Discussion

### Temperature Dependence of the Absorption Coefficient

**Figure 1:**
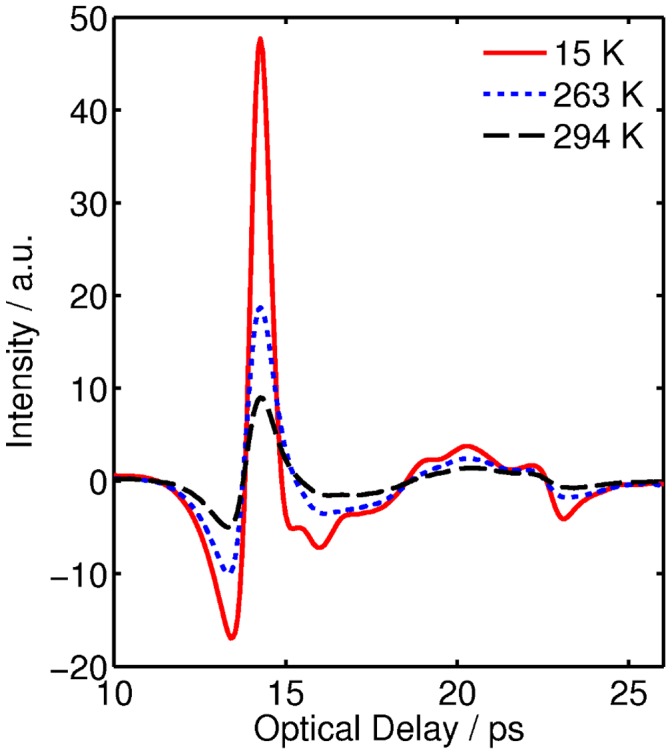
THz time-domain signals of GP2 at 15, 263 and 294 K.

**Figure 2:**
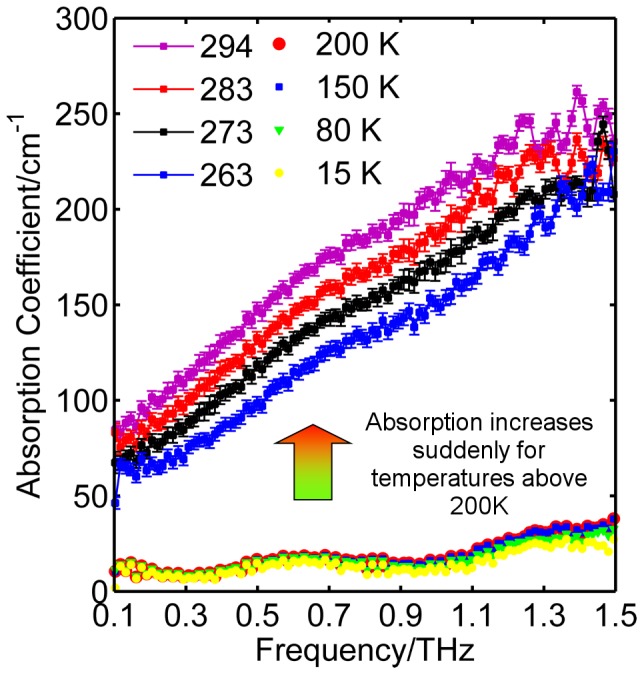
Absorption coefficient of HER2/nue for 0.1-1.5 THz at temperatures between 15 K and 294 K.

**Figure 3:**
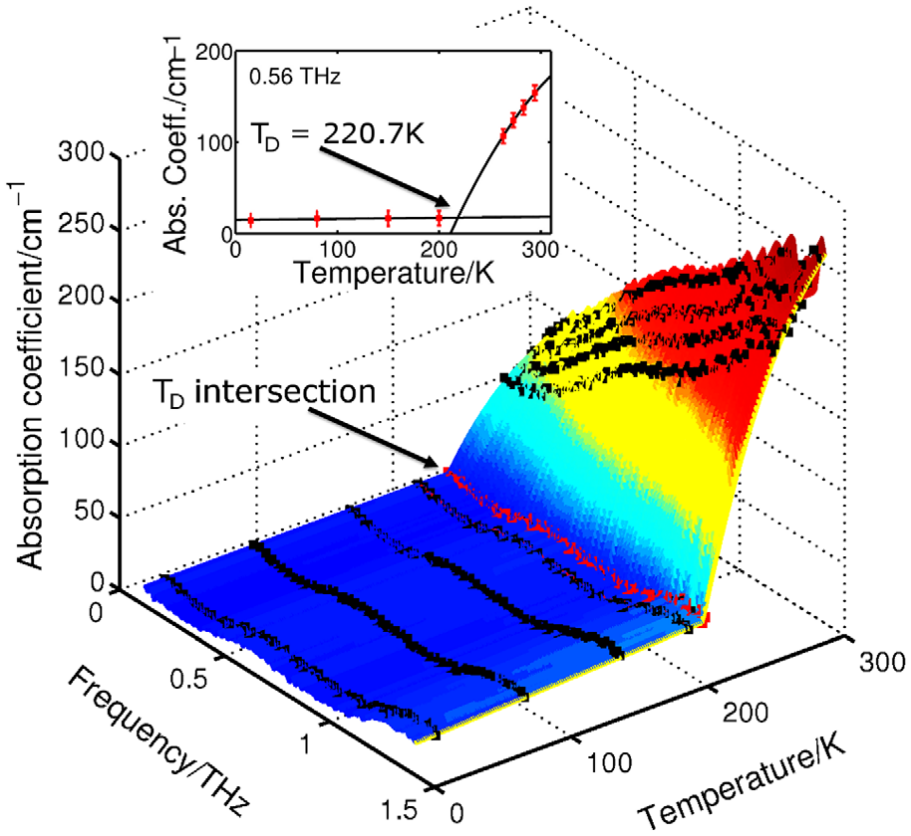
The frequency dependence of the absorption coefficient for GP2 solution at temperatures 15-294 K. The temperatures along the intersection curve give the calculated *T_D_* for each frequency, the inset shows this for 0.56 THz.

Figure 1 shows the raw THz intensity signal in the time domain for GP2 at 15 K, 263 K and 294 K. By Fourier transforming the sample data and applying Fresnel equations to both sample and reference data, as detailed in reference [19], the absorption coefficient is calculated. Figure 2 shows the absorption coefficient of GP2 in the range 0.1 to 1.5 THz at the measured temperatures between 15 K and 294 K. The error bars represent 95% confidence intervals and are too small to be seen when the temperature is below 200 K. The absorption coefficient suddenly drops between 263 K and 200 K. To obtain a full picture of how the absorption coefficient depends on both frequency and temperature, we present a 3D plot in Figure 3. The inset in Figure 3 uses the data at 0.56 THz to highlight the trend of the dependence of absorption with temperature. The data below 200 K is linearly fitted, while a quadratic curve model is adopted to describe the trend line of the points above the 200 K. Note that *T*_D_ is calculated from the intersections of the two fits for each frequency measured within the range of 0.1–1.5 THz. These data were measured in the same moisture, pressure, solution concentration, in order to focus on the changes of *T*_D_ only caused by altering frequency at each temperature.

**Figure 4:**
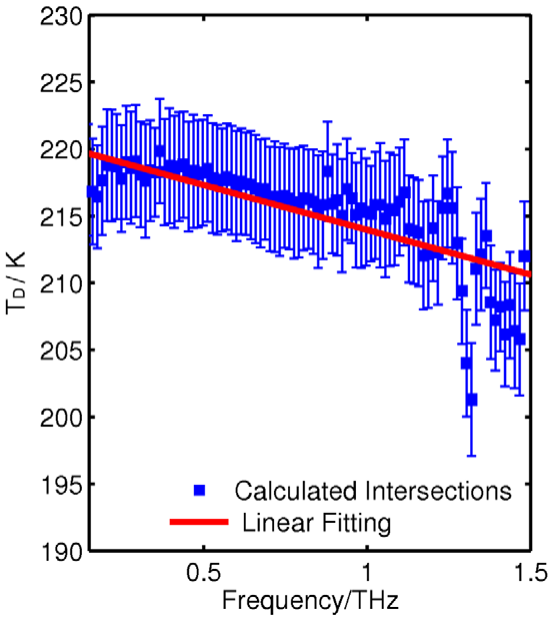
The calculated dynamical temperature *T_D_* as a function of frequency. A linear best fit is also shown (red solid line).

The intersection curve in Figure 3 is plotted in Figure 4 to highlight the frequency dependence of the dynamical temperature. The error bars in Figure 4 are the resulting intersection points from fitting the trend lines to the upper and lower 95% confidence limits of the measured data. The error is close to ±4 K across the frequency range; thus the percentage error is small at around ±2%. The measurements reveal that the dynamic transition temperature has approximately linear frequency dependence and lies between 200 and 220 K for 0.1–1.5 THz. In the next sections we discuss the molecular processes occurring in the peptide solution and how they can be modeled below and above *T*_D_. This is important for understanding how hydrated proteins function.

### Relaxation and Resonance Processes

The hydrated peptide undergoes a dynamic transition at *T*_D_, which we are able to identify by the sharp rise in the absorption coefficient. This is because the properties of the spectral absorptivity are significantly affected by the energy of the thermal radiation, as expressed by Kirchhoff’s law of thermal radiation [28]. Our measurement of pure buffer solution does not display this sharp rise, which demonstrates that the change in dynamics is due to the protein [25]. As shown in Figure 2, the absorption coefficient appears almost independent of temperature in the range 15 K to 200 K. Temperature independence is a characteristic of the vibrational density of states for processes that can be modeled by a harmonic approximation [29]. However for 263–294 K the temperature dependence is stronger and a non-linear fit (a quadratic curve) is used to fit the measured data, suggesting a non-harmonic dynamical process.

To further analyse the data we calculate the complex permittivity ε* of the protein solutions. The real (*ε′*) and imaginary (*ε′′*) parts of the complex permittivity are calculated from the frequency-dependent optical constants *n*(*ω*) and *α*(*ω*) as detailed in Equation 7 of reference [19]. The real part of the complex permittivity corresponds to the dielectric constant and the imaginary part, known as the dielectric loss factor, is a measure of the energy absorption per cycle [1].

**Figure 5:**
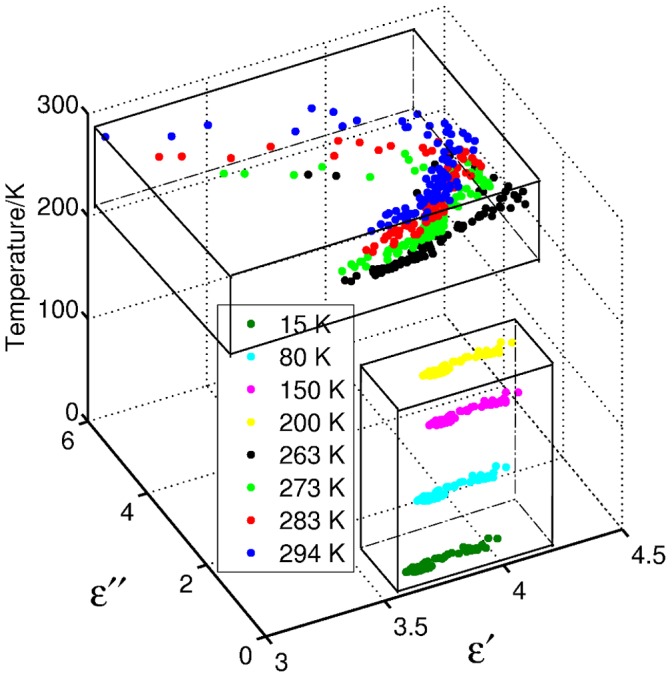
Cole-Cole plot for GP2 solution at different temperatures. The upper box indicates the distribution of the dielectric constant of the protein above *T_D_*, the lower box shows the different distribution when the temperature is below *T_D_*.

The Cole-Cole plots (*ε′* versus *ε′′*) for our data are shown in Figure 5. For a liquid with pure Debye behavior the Cole-Cole plot is a perfect semi-circular arc. If the species present in a liquid have a range of relaxation times, the Cole-Cole plot starts to deviate from a perfect semicircular arc. For GP2 we see that the data from above and below *T*_D_ are clearly separate and have different behaviors. At temperatures above *T*_D_ (263–294 K), the Cole-Cole plots are close to semicircular arcs and at temperatures below *T*_D_ (15–200 K), the data shape can be described as a skewed arc, indicating a distribution of relaxation times for these samples. Since the dielectric behavior of GP2 is different in the two temperature regions, in the next sections we consider the two regions separately and fit models to the data above and below *T*_D_.

### Modeling GP2 below *T*_D_

The function of proteins in an aqueous solution is influenced both by the spatial and dynamic arrangement of neighboring liquid layers through weak intermolecular interactions [1], [30], [31]. By using X-ray and the latest high-resolution NMR techniques, we can in general identify two types of hydration shell associated with proteins (primary and secondary). The primary hydration shell, which is also named internal water, is bound directly to protein molecules. The secondary hydration shell containing the peripheral water has a character intermediate between those of the primary shell and bulk water. Thus the water molecules from the different shells embody multiple mechanisms that can account for the nature of the protein polarization. The ubiquitous empirical Havriliak–Negami equation (Equation 1) [32] is able to describe multi-pole conditions over a range of frequencies:
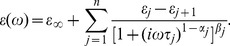
(1)Here, *ω* is the angular frequency, *n* is the number of relaxation processes, *τ_j_* are their relaxation time constants, ε_1_ = ε_s_ is the static dielectric constant, *ε_j_* are intermediate steps in the dielectric constant, ε*_n+1_* = ε_∞_ is its limiting value at high frequency. The parameters *α_j_* >0 and *β_j_* <1 describe either a symmetric or asymmetric distribution of relaxation time for process *j*. Although theoretically the peptide exists in different forms in certain solutions because of aggregation, this does not affect the physical model adopted because these molecules, no matter monomer or polymer, can be considered as dipoles in this model.

For temperatures below *T*_D_ we assume a single relaxation time can provide an adequate description and in some sense, this indicates that all dipoles (molecules) have the same environment over the time τ in a crystalline state and this enables a special case of *n = 1* in Equation 1 to be used. Based on previous studies [33], the Cole and Davison equation (with *α_j_*  = 0, 0< *β_j_* <1) hypothesizes that the formation of micro-inhomogeneities of cluster type is preferable compared to the Cole-Cole model (*β_j_* = 0, 0< *α_j_* <1) with symmetric distributions of relaxation times at temperatures below 236 K. So we use Cole-Davison equation model given by Equation 2 to determine the vibration mode of hydrated GP2 from 15 K to 200 K,
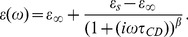
(2)


**Figure 6:**
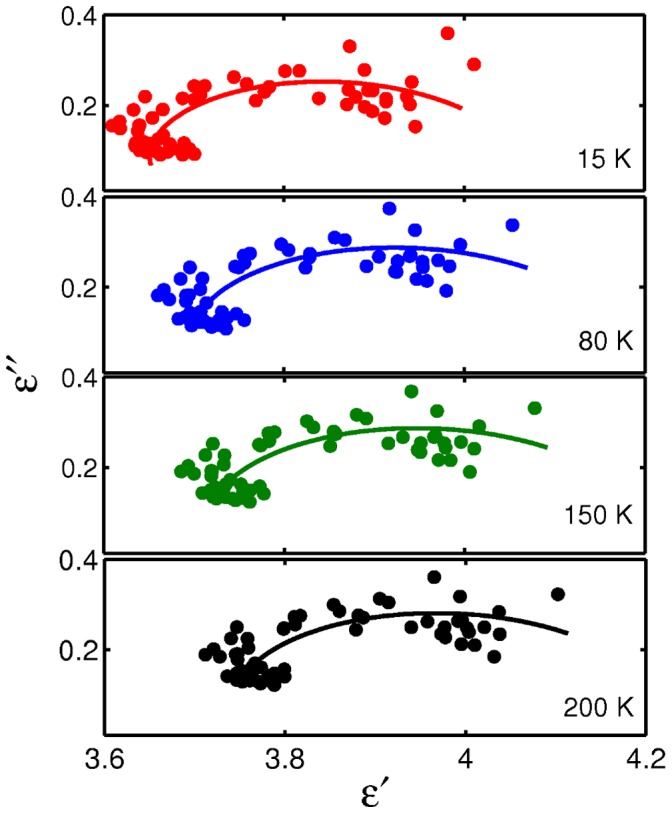
Cole-Cole plot for temperatures 15, 80, 150 and 200 K. Lines are fitted by the Cole-Davison model and have a skewed arc distribution.

Figure 6 shows the measured frequency-dependent dielectric parameters of hydrated GP2 fitted to the Cole-Davison equation at 15, 80 150 and 200 K respectively. The solid lines are calculated with the relaxation model using the parameters of Table 1. For data fitting a non-linear least squares routine is applied.

**Table 1:** Dielectric parameters of GP2 at temperatures below *T_D_* found by fitting the data to Equation 2.

Temp (K)	ε_s_	ε_∞_	τ_CD_ (*ps*)	β
15	4.43	3.58	9.61	0.61
80	4.99	3.58	22.85	0.43
150	5.27	3.55	45.41	0.55
200	5.35	3.53	48.84	0.54

### Modeling GP2 above *T*_D_

In the dielectric relaxation studies described above, a single Cole-Davison relaxation model is found to have good agreement between experimental data and the model of the polarization for 15 K to 200 K. However, when this model is adopted at higher temperatures (above *T*_D_) it fails to reproduce the experimental findings. It is suggested in reference [32] that this is usually due to the presence of an additional absorption band as well as that due to the relaxation of the orientation of the polarization, namely a multiple process should be considered in order to adequately account for the observed experimental data. This process, which is called resonance absorption, described by the Van Vleck-Weisskopf-Fröhlich type [34], is described by an exponential type of relaxation function multiplied by the cosine function with a characteristic frequency *ω*_0_:
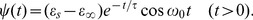
(3)


The response function corresponding to the relaxation function in Equation 3 is
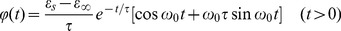
(4)and the complex dielectric function is given by Equation 5 [35].
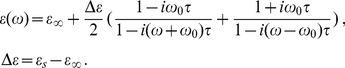
(5)Here ε_∞_ denotes the value of ε′(ω) at ω sufficiently far away from the resonance point (ω_0_). Therefore, the value of ε′(ω) on the low-frequency side becomes ε_∞_+Δε.

In the THz range, the interaction between an oscillating electric field and the protein solution system is caused by both relaxation effects and resonant absorption effects [36]. The former is due to transitions of charges or dipoles between equilibrium positions which can be described by a relaxation time τ. The resonant absorption effect is due to displacement of charges bound elastically to an equilibrium position which depends on the nature of dielectrics [37]. Thus, this process can be reasonably described by two parts, one is the dielectric relaxation based on the solvent model and the other is the resonant absorption process. The total dielectric response is then given by:
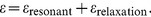
(6)


According to Debye theory, the polarization of the background ε_relaxation_ resulting from permanent dipole moments of molecules decays exponentially based on Equation 2 and a general feature of ε_resonant_ is found in Equation 5. The resulting behavior from summing these two contributions is named the Cole-Davison-Resonant-Absorption (CDRA) model. The complex permittivity for the CDRA model is given in Equation 7:
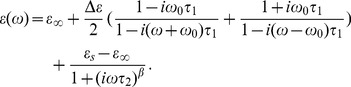
(7)


**Figure 7:**
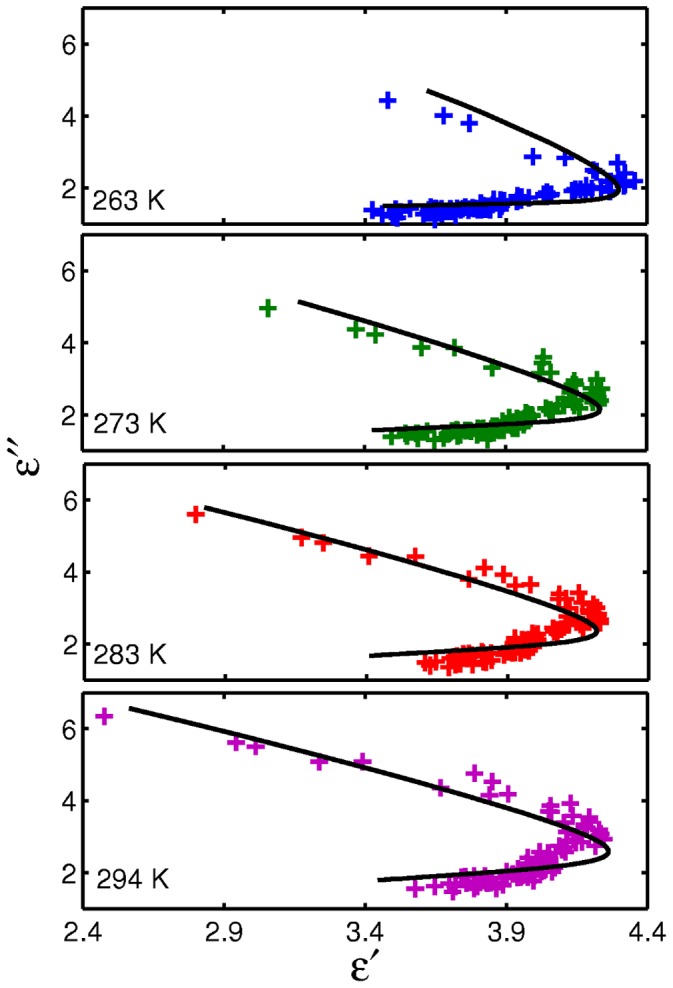
Cole-Cole plot for temperature at 263, 273, 283 and 294 K. Lines are fitted by CDRA model considering the resonance response. Note the semicircular arc distribution.

We fit our THz spectroscopy data for GP2 at temperatures above *T_D_* to this equation and the resulting parameters are given in Table 2. The data and corresponding best fits at the measured temperatures are illustrated in Figure 7. The semicircular arc distribution is clearly seen in these data.

**Table 2:** Dielectric parameters of GP2 at temperatures above *T_D_* found by fitting the data to Equation 7.

Temp (K)	ε_s_	ε_∞_	τ_1_ (*ps*)	τ_2_ (*ps*)	ω_0_/2π (THz)	β
263	29.55	10.60	74.47	0.17	3.75	0.92
273	32.58	12.09	87.29	0.18	3.59	0.82
283	32.68	12.38	89.77	0.18	3.46	0.79
294	36.89	13.92	94.76	0.18	3.44	0.77

The data show that there are two very different time scales present in the model. The first time scale τ_1_ from the resonance state is much longer and reaches nearly 95 ps, while the other timescale τ_2_, for the relaxation step, is almost 500 times faster. Since the driving force arising from the shell of water around the protein can induce dynamical behavior [23], it is necessary to understand relaxation processes in water. Many dielectric relaxation studies on bulk water have identified two relaxation times (slow and fast) for the polarization: at room temperature the slow one is about 8 ps and the fast one is 0.18 ps [38]–[42].

**Figure 8:**
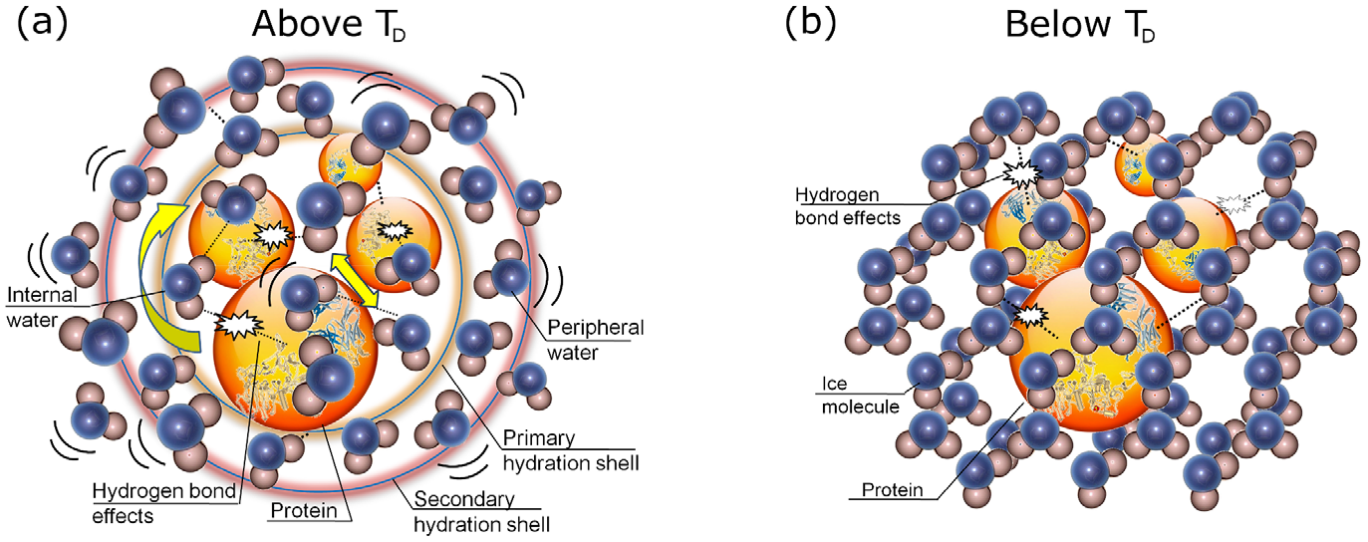
Schematic diagrams to illustrate the molecular processes above and below *T*_D_. (a) Both slow (τ_1_) and fast (τ_2_) dynamical processes were present in the protein solution when the temperature is above *T_D_*. The slow process is from the resonance absorption of the hydrated protein; the fast process was caused by the rearrangement of hydrogen bonding within the peripheral water. (b) Only one slow relaxation process τ_CD_ was identified at temperatures below *T_D_*.

The fast process in water has been attributed to the reorientation of individual water molecules, and the slow process has been attributed to the re-orientation of a several hydrogen molecules for example in a pentomer structure [40]. We therefore attribute the temperature independent fast relaxation time in GP2 to the reorientation of individual water molecules in the peripheral water. The large molecular weight of the protein molecule relative to the water molecule will cause much slower processes. From the data in Table 2, we also notice that there is a single resonance absorption at 3.44–3.75 THz. We attribute this to the displacement of bound charges within the hydrated protein from their equilibrium positions and predict that the power loss in the system will have a maximum near this frequency [43]. Though this frequency is beyond the bandwidth of our measurement system, the characteristic frequency *ω*_0_ (in Table 2) decreases with increasing temperature, showing “red shift” behavior. Such red shift behavior is indicative of anharmonicity of the vibrational potential. The molecular origin of these processes is illustrated in Figure 8.

### Combining the Molecular Processes above and below *T*_D_

**Figure 9:**
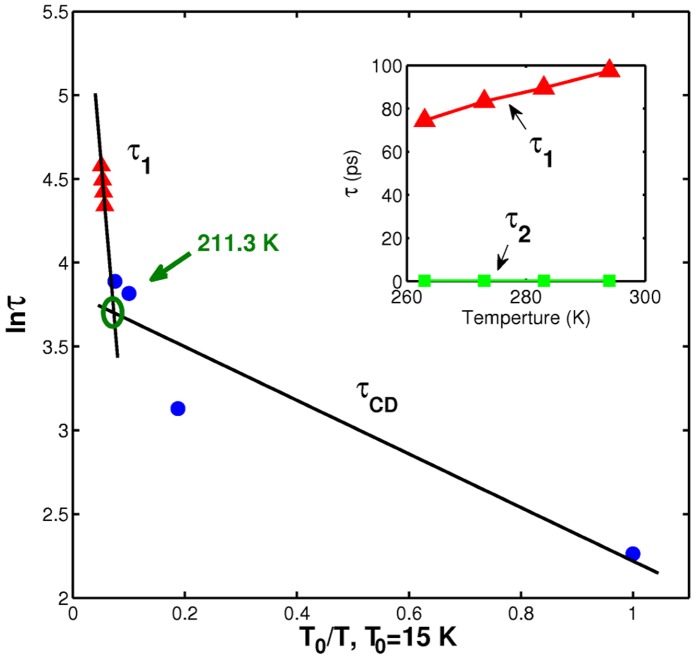
The temperature dependence of the fitted relaxation times τ_CD_ and τ_1_ plotted using ln(τ) vs. T_0_/T scale (T_0_  = 15 K). The corresponding fits to the Arrhenius law crossover at 211.3 K. The inset shows the temperature dependence of the fast and slow relaxation times for temperatures above *T_D_*.

To combine the dynamical behavior of the GP2 across the whole range of temperatures measured, we plot the fitted time constants relating to the slow processes τ_CD_ for below *T*_D_ and τ_1_ for above *T*_D_ versus relative temperature (*T*_0_/*T*) in Figure 9. For all the studied temperatures τ_1_ (fitted using the CDRA model) and τ_CD_ (from the adopted Cole-Davison model) both show a gradual decrease. In each case, the behavior of the hydrated protein is close to an Arrhenius law (lnτ ∝ 1/*T*), black solid lines), revealing a dominant term [44] in the molecular dynamics occurring both above and below the dynamic temperature. As per the analysis above, this dominant term is related to the rearrangement of the hydrogen bonding between the protein and the internal water. Thus internal water, considered an integral part of a protein structure, strongly affects the dynamical properties of the protein. The crossover point in Figure 8 denotes that the dynamic transition occurs at 211.3 K, which matches well with the onset of non-harmonic motion of the protein [23], which we previously deduced to occur between 200 and 220 K. In addition to the slower resonant process time above the dynamic temperature, the inset in Figure 8 indicates the detail of the vibration process related to fast relaxation times τ_2_. These parameters fitted by the CDRA model indicate that the resonance absorption response can be affected by the dynamic process but is not an “interaction” behavior *per se*.

### Limitations of Study and Future Work

This is the first THz study of the dynamical temperature transition of hydrated GP2: it is a fundamental study and demonstrates the capabilities of THz spectroscopy as well as highlighting that GP2 has interesting features in the THz range. However, there are a number of limitations in this study that now motivate further work for building upon this research. The concentration of the peptide used in this study is much higher than present in human saliva – further work is needed to investigate the solubility and concentration dependence of the THz absorption as well as the specificity to GP2. For instance, the formation of aggregates including the degree of polymerization and formation of micelles should be investigated as well as sequence dependence.

### Conclusion

In this paper we have shown for the first time that GP2 undergoes a frequency dependent temperature transition at 200–220 K at 0.1–1.5 THz. By fitting suitable molecular models to the frequency response we determined the molecular processes involved above and below the transition temperature. Below *T*_D_ the dynamic transition is dominated by a simple harmonic vibration with a slow and temperature dependent relaxation time constant (τ_CD_) relating to the hydrogen bonds between the protein and the peripheral water. Above *T*_D_, the dynamic behavior is governed by two oscillators, one of which has a fast and temperature independent relaxation time constant (τ_2_) relating to the reorientation of individual water molecules surrounding the protein and the other of which is a heavily damped resonance with a slow and temperature dependent time constant (τ_1_) relating to the hydrogen bonds between peripheral water molecules and the protein molecule. We fit the Arrhenius equation to the slow timescale processes using τ_CD_ below *T*_D_ and τ_1_ above *T*_D_. The crossover point of the fit is due to the onset of non-harmonic motion and this occurs at *T*_D_ ∼211 K, which is consistent with our frequency dependent estimate of 200–220 K.

Our measurements and modeling of GP2 highlight the unique capabilities of THz spectroscopy for protein characterization. This provides motivation for further investigation into the potential to use THz spectroscopy of HER2/neu and its derivatives as a potential cancer screening testvia saliva in the future.
